# Biomarkers of Cervical Dysplasia and Carcinoma

**DOI:** 10.1155/2012/507286

**Published:** 2011-10-29

**Authors:** Sonya J. Hwang, Kenneth R. Shroyer

**Affiliations:** ^1^Department of Pathology, Hospital Level 2, Room 766, Stony Brook University Medical Center, Stony Brook, NY 11794-7025, USA; ^2^Department of Pathology, Basic Science Tower, Level 9, Stony Brook University Medical Center, Stony Brook, NY 11794-8691, USA

## Abstract

Although cervical cytology screening has decreased the incidence of cervical cancer in industrialized countries, HPV-related cervical disease, including premalignant and malignant lesions, continues to represent a major burden on the health care system. Some of the problems include the potential for either under- or overtreatment of women due to decreased specificity of screening tests as well as significant interobserver variability in the diagnosis of cervical dysplastic lesions. Although not completely elucidated, the HPV-driven molecular mechanisms underlying the development of cervical lesions have provided a number of potential biomarkers for both diagnostic and prognostic use in the clinical management of these women.

## 1. Introduction

Cervical cancer remains a leading cause of morbidity and mortality worldwide, with an estimated incidence of 470,000 [1]. Approximately 230,000 women die each year from cervical cancer; over 190,000 of these women are from developing countries in South America, sub-Saharan Africa, and the Far East [2]. In the United States, the incidence of invasive cervical cancer is much lower; the American Cancer Society estimated that in 2010, there were approximately 12,200 new cases, with the number of estimated deaths at 4,210 [3]. The differences in incidence are attributed mainly to the utilization of cytological screening in numerous industrialized countries during the latter half of the 20th century [4]. In the US, the main burden of cervical disease manifests as a much higher number of premalignant lesions, including low grade cervical intraepithelial neoplasia (CIN1) (over 1.4 million new cases) and high grade lesions (CIN2/3) (330,000 new cases) [5]. Overall, the clinical management of patients with cervical premalignant and malignant lesions represents a significant burden on the health care system. Although improved methods are needed to improve the accuracy of cervical cancer screening, it is also important to consider that the vast majority of cervical cancer deaths worldwide occur in women that have never been screened.

The association between cervical premalignant and malignant epithelial lesions and human papillomaviruses (HPV) has been well established [6, 7]. There are over 100 defined HPV types, and these have been subdivided into high-risk (HR-HPV) and low-risk (LR-HPV) categories, based on their association with cervical cancer [8, 9]. Although the majority of women with HR-HPV infections have only transient infections that do not lead to malignant transformation of the cervical mucosa, HR-HPV is the etiologic agent of virtually all cases of cervical cancer. Dunne et al. found that while the overall prevalence of HPV infection (including both low-risk and high-risk types) in US women between the ages of 14 and 59 years of age was 26.8% (*n* = 1921), the prevalence of high risk HPVs was 15.2%. Furthermore, there was a marked peak in HR-HPV infection in women between the ages of 20 and 24, with a prevalence of 29% [10]. The vast majority of HPV infections (up to 90%) regress spontaneously, without treatment, after a few months [11, 12]. If the viral infection persists, however, the risk of developing a precancerous lesion increases as well as the risk of developing an invasive carcinoma [12, 13]. This underscores the importance of accurate diagnosis as well as identification of those lesions at highest risk for progression. 

Histological examination of colposcopy-guided biopsies is still considered the “gold standard” in the assessment of cervical lesions; however, the histologic assessment of these lesions is limited to the interpretation of the morphology, with little to no information regarding the risk of persistence, progression, or regression. In addition, histologic assessment of cervical lesions is complicated by interobserver variability [14]. The main interpretive categories include distinguishing normal from dysplasia (CIN) of any grade and low-grade (CIN1) lesions from high-grade (CIN2/3) lesions. Errors in histologic diagnosis lead to either overtreatment of patients who will not benefit from intervention or, conversely, under-treatment of patients with clinically significant high-grade lesions that received false negative diagnoses. The HPV life cycle and molecular events leading to cellular transformation, while not completely elucidated, have provided insight into potential biomarkers that can be used as adjunctive tests to improve diagnostic accuracy of cervical lesions as well as, identify those patients at risk for progression to cancer. This paper focuses on those biomarkers that appear to be most relevant in the clinical management of patients with HPV-related cervical disease.

## 2. HPV Review

Human papillomaviruses (HPVs) are a diverse group of viruses (numbering more than 100) that can infect numerous epithelial sites and cause a variety of epithelial lesions, including common warts, verrucas, laryngeal papillomas, and genital condylomata, depending on the type of HPV [15]. The different types that infect the female genital tract have been divided into high-risk types (HR-HPV, including types 16, 18, 26, 31, 33, 35, 39, 45, 51, 52, 53, 56, 58, 59, 66, and 68) and low-risk types (LR-HPV, including types 6, 11, 40, 42, 54, and 57). The LR-HPVs are associated with benign exophytic genital warts (condylomata acuminata) and are rarely associated with high-grade squamous intraepithelial lesions (HSILs) or invasive squamous cancers. Conversely, HR-HPVs, especially HPV-16, the most prevalent virus infecting the cervix, are associated with the entire spectrum of CIN lesions as well as, invasive squamous carcinomas. Recent studies have demonstrated that HR-HPV types account for almost 90% of all cervical infections [16].

The HPV genome consists of a double-stranded circular genome that includes early and late open reading frames (ORFs). The early ORFs E1 through E7 encode proteins that are involved in the regulation of DNA replication and cell proliferation, while the late ORFs L1 and L2 encode the two viral capsid proteins [15]. CIN1 lesions reflect high levels of HPV episomal replication, the so-called “productive infections.” In these cases, the E1/E2 open reading frames serve as negative regulators of E6 and E7. In CIN 2 or more severe lesions (CIN2+), however, arrested squamous maturation no longer supports effective HPV DNA replication, and the copy number of HPV DNA is generally low. In these lesions, a transforming event, often associated with disruption of E1/E2 by the integration of the HPV genome into the host genome, results in the unregulated expression of E6 and E7. Overexpression of E6 promotes cell-cycle progression by promoting degradation of p53, allowing cell-cycle progression even in the face of genomic damage, while E7 promotes the degradation of Rb, resulting in the release of transcription factor E2F and cell-cycle progression. The degradation of Rb also results in the hypomethylation of the p16^INK4a ^ promoter, enabling high-level overexpression of p16^INK4a ^ [17]. The identification of these major components in HPV-mediated oncogenesis provides potential targets for clinically relevant biomarkers.

## 3. HPV DNA 

The most widely used and extensively investigated biomarker in the management of cervical disease is HPV DNA testing. There are a wide range of HPV detection techniques, including in situ hybridization, and genotyping assays, including molecular amplification assays with or without genotyping [17]. The *Digene* HPV test, which uses Hybrid Capture 2 (HC2) technology, and the Cervista HPV HR assay are the only methods that currently have FDA approval for diagnostic testing in the United States. 

The *Digene* HPV Test (Qiagen, Valencia, Calif, USA) was the first HPV test that was licensed by the FDA (United States Food and Drug Administration). This test is a solution-phase hybridization assay that uses RNA probes complementary to HPV DNA, resulting in signal amplification. This test detects the presence of 13 HR-HPV types (16/18/31/33/35/39/45/51/52/56/58/59/68) or 5 low-risk types (6, 11, 42, 43, and 44). The assay is usually performed using only the HR-HPV probe set, since LR-HPV is not clinically significant. In the ASCUS-LSIL Triage Study (ALTS), HC2 was shown to provide more effective triage of ASCUS cytology than a repeat cytology examination [5]. Other randomized large studies have reported that 50% to 70% more precancerous lesions may be diagnosed when HPV testing is incorporated in primary screening [18–20]. Another advantage is that because a negative result excludes the risk of HPV-related disease in subsequent years, screening intervals may safely be increased to 3 to 5 years in those patients with a negative result. Other advantages include good interlaboratory reproducibility [21] and ease of use. One of the disadvantages is that this assay does not produce information on individual HPV types; instead, the presence of at least one of the high-risk or low-risk types is reported. This is a significant limitation, since persistent infection with HR-HPV is a risk factor for progression to cervical cancer and with the advent of HPV vaccines, it is increasingly relevant to perform HPV genotyping to identify oncogenic HPV vaccine types [22]. HPV genotyping is of clinical interest, since the risk of developing a precancerous lesion is between 10%, and 15% with HPV types 16 and 18, and below 3% for all other high-risk types combined. Genotyping information could provide more information regarding risk-stratification as well as persistence of infection [23].

The Cervista HPV HR test (Hologic, Bedford, Mass, USA) detects the presence of 14 HPV types designated as high risk by the International Agency for Research on Cancer (IARC), consisting of 16/18/31/33/35/39/45/51/52/56/58/59/66/68. This assay utilizes Invader chemistry, a signal amplification method for the detection of specific nucleic acid sequences. This method comprises two isothermal reactions: a primary reaction on the targeted DNA sequence and a secondary reaction that produces a fluorescent signal. In a comparison between the Digene and Cervista assays, the Cervista assay demonstrated 100% sensitivity in the detection of CIN 3 or worse and 98% sensitivity for the detection of CIN 2 or worse [24]. After adjustments to compensate for potential bias related to availability of biopsy histologic diagnoses, the expected clinical performance of the HPV HR test is 95.49% sensitivity, specificity 63.3%, PPV 10.1%, and NPV 99.7%. In this study, the authors reported that the Cervista assay had a lower false-positive rate compared to the Digene assay (attributed to cross-reactivity with low-risk HPV types). Some other strengths of the Cervista assay include an internal positive control to determine the presence of sufficient DNA and the presence of potentially interfering substances and requiring a smaller sample volume compared to other assays. However, IARC determined that there is limited evidence to conclude HPV 66 is carcinogenic; although the prevalence of HPV 66 in women is low, this reclassification may have a marginal impact on the false-positivity rate [25]. Also, similar to the Digene assay, information regarding individual HPV types is not provided. To address this, a DNA-based genotyping assay was developed as well. 

The Cervista HPV 16/18 test (Hologic, Bedford, Mass, USA) has been approved by the FDA for use in conjunction with the Cervista HPV HR test. This test utilizes the same Invader chemistry used by the HPV HR test in the analysis of cervical cytology specimens. Clinical validation and analytical performance studies report that the Cervista HPV 16/18 genotyping test demonstrated a high degree of analytical sensitivity, and specificity, and performed as expected in women with ASC-US cytology who were positive for HR HPV [25, 26]. These studies support the utilization of the genotyping test in the proper clinical context.

The polymerase chain reaction (PCR) method of detecting HPV enables the sensitive amplification of even small amounts of HPV DNA, enabling the evaluation of extracts of formalin-fixed histologic sections which generally yield fragmented DNA [17]. Briefly, the two major types of available PCR assays are type-specific and consensus sequence assays. The type-specific assays amplify a single HPV genotype, necessitating multiple separate PCR assays and increasing the cost for genotyping each sample. The consensus assays detect a wide range of HPV types, most commonly using primers that target the L1 region. Once the sample is amplified, there are a number of methods that may be used to determine the specific type of HPV, including nucleic acid hybridization, restriction fragment length polymorphism, and sequencing. There are, however, cost and other considerations currently that limit clinical application.

Overall, HPV DNA testing has a sensitivity above 90% for the detection of underlying CIN2+ lesions but has generally poor specificity for underlying clinically significant lesions, because most positive cases represent only transient infections rather than providing evidence of cervical mucosal transformation [27]. As a result, HPV testing is useful for the triage of women with ASCUS cytology but is generally not used for the triage of women under the age of 30 with other cytologic diagnostic test results. HPV testing in women over the age of 30, in combination with liquid-based cervical cytology, can, however, be used to increase the screening interval in women over age 30 due to the high negative predictive value of HPV testing. Although HPV genotyping provides more information regarding a patient's risk for progression, individual typing assays are not commonly used for routine cervical cancer screening.

## 4. HPV Viral Load

Viral load may be a useful marker in predicting the risk of progression. High viral load is often considered to be indicative of persistent infection and progression, while low viral load has been interpreted to reflect HPV viral clearance. A fundamental pitfall of this concept, however, is that CIN1 lesions reflect productive infections and may have thousands of viral copies/cell in upper layers of the cervical mucosa, but CIN2/3 and SCC lesions may have as low as a single copy of viral DNA/cell (commonly integrated into the host genome but not supporting viral replication) [28]. Thus, there is at least a theoretically increased risk of false negative HPV test results in high-grade lesions compared to low grade lesions, unless the high grade lesional cell sample also includes cells that are derived from a coexisting low-grade productive infection. HPV viral copy number may be determined using PCR assays that target type-specific HPV DNA and normalize to the total human DNA present [29]. In a study by Carcopino et al., HPV 16 and 18 viral loads were related to the severity of the cervical lesion [30] although as suggested above, these results may have been impacted by the presence of cells that were actually derived from low-grade lesions.

## 5. HPV mRNA

Assays for the detection of E6/E7 mRNA have been developed based on the concept that E6/E7 expression results in a transforming event with unregulated cell-cycle progression due to degradation of p53 and Rb [31]. The PreTect HPV-Proofer assay (NorChip AS, Klokkarstua Norway) is a commercially available assay (in Europe only) to detect E6/E7 mRNA from five HR-HPV types (16, 18, 31, 33, and 45) [32]. A positive HPV-Proofer result is indicative of E6/E7 integration and identifies a high risk of persistent infection. In a study by Molden et al., a comparison of HPV DNA and mRNA was performed on women with an initial diagnosis of ASCUS or LSIL on cervical cytology, with a 2-year follow-up period [33]. In this study, women with a positive HPV-Proofer assay were approximately 70 times more likely to be diagnosed with CIN2 or greater than women who tested negative. Consensus PCR testing for HPV was also performed; women who tested positive were 6 times more likely to be diagnosed with CIN2 or greater than women who tested negative. These results suggest that the HPV-Proofer assay is as sensitive but is more specific than HPV PCR for the detection of underlying high-grade lesions. 

The APTIMA HPV assay is another commercially available test for mRNA detection (Gen-Probe, San Diego, Calif, USA). The APTIMA HPV assay detects mRNA from 14 HR-HPV types (16, 18, 31, 33, 35, 39, 45, 51, 52, 56, 58, 59, 66, and 68) using liquid-based cervical cytology specimens. The assay involves target capture, target amplification, and the detection of the amplification products. This assay is currently in clinical trials, with a focus on the identification of women that are at high risk for persistent infection.

## 6. HPV L1 Capsid Protein

The L1 capsid protein represents approximately 90% of the total protein on the virus surface and is generally detectable during the reproductive phase of HPV infection. The L1 protein is abundant in productive infections [34, 35]; conversely, it is found only in rare cases of CIN3, and it is not produced in carcinomas [34]. In general, CIN2/3 lesions are unlikely to support productive HPV infection, because viral maturation depends on squamous maturation that, by definition, is arrested in CIN2/3. There have been a few studies evaluating the prognostic significance of L1 status; although the studies are relatively small, it has been suggested that L1 status may have utility in the prediction of disease progression [35–37]. A recent study by Galgano et al., however, found that L1 was neither sensitive nor specific for the detection of CIN2/3 lesions [38].

## 7. p16^INK4a ^ and Ki-67

p16^INK4a ^ is a tumor-suppressor protein and cyclin-dependent kinase (cdk) inhibitor that blocks cdk4- and cdk6-mediated pRb phosphorylation to inhibit E2F-dependent transcription and cell-cycle progression [39]. In most cervical carcinomas, the functional inactivation of pRb by HPV E7 results in the overexpression of p16^INK4a ^ and the accumulation of the protein in cells. p16^INK4a ^ is thus a surrogate marker of HPV E7-mediated pRb catabolism, providing evidence of transformation of the cervical mucosa [40, 41]. p16^INK4a ^ has been successfully deployed for the classification of HPV-related disease for several reasons [42]: (1) the expression of p16^INK4a ^ is directly linked to the HPV oncogenic action, since continuous expression of E7 is necessary to maintain the malignant phenotype, (2) the expression of p16^INK4a ^ is independent of the HPV type, and therefore, genotyping does not need to be performed, and (3) the expression of p16^INK4a ^ by cycling cells is a specific marker of HPV-E7 overexpression or other events that inactivate Rb [43]. Immunohistochemical analysis has demonstrated that diffuse staining for p16^INK4a ^ is present in almost all cases of CIN2, CIN3, and squamous cell carcinoma (as well as in endocervical glandular neoplasia); however, it is rarely detected in benign squamous mucosa or CIN1 lesions associated with LR-HPV [40, 41]. One limitation of the analysis of p16^INK4a ^ as a marker of cervical neoplasia is that focal and occasionally diffuse expression can also be observed in benign endocervical intercalated columnar cells, in tuboendometrial metaplasia, and in cervical endometriosis [44]. The expression of p16^INK4a ^ in these cells, however, denotes no premalignant potential. Focal staining can also be detected in the lower third of some CIN1 lesions and in the upper third of the epithelium in a few cases of squamous metaplasia. The diffuse pattern of p16^INK4a ^ expression within the lower third of the squamous mucosa, however, is highly specific for CIN1+ lesions, and diffuse expression in glandular epithelial cells usually reflects endocervical glandular neoplasia (lesions with some but not all features of AIS), AIS, or invasive adenocarcinoma.

For cervical tissue punch and cone biopsies, immunohistochemistry for p16^INK4a ^ has been reported to reduce interobserver disagreement when compared with diagnosis of H&E stained sections [45–47]. In one study, 496 cervical histology H&E-stained slides (each representing an independent case, either punch or cone biopsy) were evaluated by 6 pathologists. Interobserver agreement for punch biopsies was moderate (mean *κ* = 0.49) and substantial for cone biopsies (mean *κ* = 0.63) [45]. The addition of p16^INK4a ^-immunostained, consecutive slides read together with the H&E-stained slides significantly improved the interobserver agreement for the interpretation of both punch and cone biopsies. For the punch biopsies, the *κ* value increased from 0.49 (moderate agreement) to 0.64 (substantial agreement), and the *κ* value for the cone biopsies increased from 0.64 to 0.70. A subsequent study by Bergeron et al. [48] addressed the utility of p16^INK4a ^ testing for both increasing interobserver agreement as well as increasing diagnostic accuracy. In this study, H&E-stained slides from 500 cases (comprising cervical punch and cone biopsies) were interpreted by twelve community pathologists. These interpretations were compared to the “gold standard” diagnoses established by three expert gynecologic pathologists. After a “washout” period of at least four weeks, the same H&E-stained slides were reassessed by the twelve pathologists, but this time in conjunction with p16^INK4a ^-immunostained matched slides. The pathologists were blinded to their original diagnoses as well as the gold standard diagnoses. Overall, diagnostic accuracy for high-grade CIN was significantly improved with the addition of p16^INK4a ^-immunostained slides. The mean sensitivity increased from 0.77 to 0.87 (an increase in sensitivity of 13%). The number of missed high-grade CIN cases was reduced by 45%. The number of cases with a gold standard diagnosis of CIN 3 that were missed by the community-based pathologists was decreased by 60%. Importantly, this gain in sensitivity was not associated with a relevant loss in specificity. Also, the interobserver agreement of the community-based pathologists for categorizing lesions as high-grade CIN versus CIN 1 or negative for dysplasia significantly improved with the addition of the p16^INK4a ^-immunostained slides, with a *κ* coefficient of 0.749 (for H&E stained slides only, the *κ* coefficient was 0.566). Also demonstrated in this study was the relative ease with which accurate, reproducible interpretation of p16^INK4a ^-immunostained slides can be implemented into clinical practice [48]. In addition to improving diagnostic accuracy and reproducibility, the use of p16^INK4a ^ immunohistochemistry may help in identifying CIN1 lesions that are associated with HR-HPV types; these lesions are at an increased risk for progression to high-grade dysplasia or carcinoma [49].

p16^INK4a ^ has also recently emerged as a sensitive and specific diagnostic adjunct for underlying CIN2+ lesions in cervical cytology specimens [50, 51]. Most studies that have evaluated the use of p16^INK4a ^ as an immunocytochemical diagnostic adjunct have relied on the use of scoring criteria that depend on both the morphologic interpretation of p16^INK4a ^ positive cells and on the use of quantitative thresholds to establish positive test results [50]. Samarawardana et al. established rigorous criteria for scoring p16^INK4a ^ test results that was associated with decreased sensitivity and negative predictive value but improved specificity and positive predictive value compared with most of the previous reports of p16^INK4a ^ test performance. Denton et al. used a different scoring system in the evaluation of p16^INK4a ^ test results and also demonstrated that the use of p16^INK4a ^ immunostaining on cytology provides significantly better specificity than HR-HPV for the triage of ASC-US and LSIL cytology cases [51]. It is important to emphasize that the primary value of p16^INK4a ^ and other cervical cancer biomarkers is to improve test specificity rather than sensitivity relative to HPV testing.

Ki-67 is a proliferation marker that is confined to the parabasal cell layer of normal stratified squamous mucosa but shows expression in the stratified squamous epithelium in CIN lesions in correlation with the extent of disordered maturation. Although Ki-67 has been used as a diagnostic adjunct for the classification of cervical tissue specimens [52, 53], the expression of Ki-67 alone does not discriminate HPV-mediated dysplasia versus benign proliferating cells in benign reactive processes, which limits its use in cytologic specimens as a specific marker of underlying CIN or glandular neoplasia. Recent large-scale studies from Europe and pilot studies from the US, however, show that a dual stain approach for p16^INK4a ^ and Mib-1, (using the CINtec Plus kit from MTM Laboratories, Westborough, Mass, USA) can be used to score cases positive on the basis of a single dual stained epithelial cell, independent of cell morphology, resulting in sensitivity that rivals HPV testing but with specificity that is greater than that provided by HR-HPV testing [54–60].

## 8. DNA Aneuploidy

HPV infection may lead to DNA hypermethylation (discussed below), disruption of the normal cell cycle, and chromosomal aberrations, all of which may lead to changes in DNA content. Studies using DNA-cytometry of Feulgen-stained cytology material to assess ploidy have demonstrated significant differences in aneuploidy between HSILs and LSILs: 79% aneuploid versus 4%, respectively [61]. Subsequent studies have reported a strong association between highly aneuploid squamous cells and HR-HPV [62] as well as a positive predictive value of 81.8% for CIN 2 for 9c cells [63]. A prospective study by Grote et al. demonstrated a significant increase in DNA aneuploidy in cervical cytology material from patients with CIN 1 (54%) and CIN 2 (64.3%) to CIN 3 or greater (83.3%) on subsequent biopsies [64]. In a preliminary retrospective study assessing the utility of DNA ploidy in the management of ASC cytology specimens, Lorenzato et al. suggest that the combined use of HR-HPV testing and DNA ploidy measurement on ASCUS cytology specimens may improve the triage of women who have to undergo colposcopy as well as identify patients with a diagnosis of ASC-H at higher risk for CIN 2 or greater lesions [65]. DNA image cytometry has become increasingly standardized and represents an objective and highly reproducible diagnostic procedure [64].

## 9. ProExC Test

The ProExC test (BD TriPath Imaging, Burlington, NC, USA) is a recently developed immunocytochemical assay for the detection of minichromosome maintenance proteins (MCMs) and in previous formulations, Topoisomerase 2*α*, in cervical cytology slides, as a marker of aberrant S-phase induction and underlying high-grade dysplasia. MCMs are members of the DNA licensing factor family that are required for the origination of DNA replication and are overexpressed in cervical high-grade dysplasia and carcinoma [66–68]. Preliminary studies demonstrated that the ProExC test is consistently positive in HSILs and negative in normal cytologic specimens [69, 70] but to date, the ProExC assay has not come into widespread utilization as a diagnostic adjunct for cervical cytology due at least in part to expression of MCMs in some benign cycling squamous and glandular cells [71, 72]. Further confirmation of the performance of this test as a diagnostic adjunct for cervical cytology will depend on the results of large-scale trials including biopsy correlation and clinical outcome correlation.

## 10. Methylation Markers

As part of the search for novel and relevant biomarkers in cervical disease, attention has been focused on methylated genes. Of particular interest is that p16^INK4a ^ has been found to be inactivated in numerous cancers due to mutations and epigenetic alterations (patterns of altered gene expression mediated by mechanisms that do not affect the primary DNA sequence) [73]. Methylation of a CpG island within the p16^INK4a ^ exon1*α* has been associated with a variety of malignant tumors, such as nonsmall cell lung cancer, colorectal cancer or pancreatic cancer [74–76]. Several groups have analyzed cervical cancers for p16 exon1*α* methylation, with frequencies ranging from 19% to 61% [77–84]; however, most of these studies reported methylation data without confirmation of the expression level. Nehls et al. performed a detailed analysis of p16^INK4a ^ exon1*α* methylation, with comparison to p16^INK4a ^ expression, using both cell lines and clinical samples [85]. They found composite or complete methylation of p16^INK4a ^ exon1*α* without any influence on p16^INK4a ^ expression and concluded that methylation in this region does not suppress p16^INK4a ^ expression. Wentzensen et al. recently published a systematic literature review of studies analyzing the utility of methylation markers in cervical cancer [86]. They identified 51 studies analyzing 68 different genes for methylation across all stages of cervical carcinogenesis. This group found that the published data was highly heterogeneous; for 7 genes, there was a reported range of methylation frequencies in cervical cancers of greater than 60% between studies. They did identify 3 markers, DAPK1, CADM1, and RARB, which showed elevated methylation in cervical cancers consistently across studies. Thus, based on these findings, no methylation markers can yet be utilized in cervical cancer screening or triage settings. Similar to other diagnostic molecular approaches, large, well-powered epidemiologic studies are still needed to identify and validate candidate methylation markers of cervical neoplasia.

## 11. FISH

Fluorescent in situ hybridization (FISH) technology has increasingly been recognized as a valuable tool to evaluate cervical dysplasia [87, 88]. Studies have demonstrated that one of the most consistent chromosomal abnormalities identified in cervical carcinoma is gain of chromosome arm 3q, which is detected in approximately 70% of cervical carcinomas [89, 90]. These extra copies result in a gain of the human telomerase RNA gene (TERC) located in the 3q26 region. The gene product, telomerase, is involved in chromosome maintenance by providing telomere stability and regulating telomere length. In a study by Caraway et al., FISH analysis for gain of chromosome 3q was performed on cervicovaginal liquid-based preparations, and results were compared with cytologic diagnosis and concurrent/subsequent biopsies [91]. Patients with HSIL or squamous cell carcinoma cytologic diagnoses had significantly higher percentages of cells with 3q26 gain than patients with negative or ASC-US diagnoses. Seppo et al. demonstrated that a fully automated FISH scoring system can detect gain of 3q in liquid cytology samples [92]. Another study evaluated HPV DNA and telomerase using a different assay (telomeric repeat amplification protocol, TRAP) as diagnostic adjuncts in cervical cytology specimens [93]. Interestingly, telomerase showed a low sensitivity (29.9%) for biopsy-confirmed CIN 2/3; the study concluded that the TRAP assay for telomerase is unlikely to be used as a diagnostic adjunct. FISH analysis for 3q appears to hold more promise as a useful biomarker. 

## 12. Conclusion

Despite the tremendous progress that has been achieved in the screening and management of women with HPV-related cervical disease, there is still a need for clinically robust biomarkers to further refine the screening, triage, and management of women. In this paper, we focused on those biomarkers that have the greatest utility in the clinical setting, such as those that will increase screening and diagnostic accuracy of cervical specimens and tissue biopsies, and we provide information regarding the risk for progression to a more severe lesion. Examples of these include HPV DNA testing for the effective triage of women with abnormal cervical cytology, and the use of p16^INK4a ^ immunohistochemistry to increase diagnostic accuracy of dysplastic lesions. Merely identifying the presence of HPV infection is not sufficient, as multiple studies have demonstrated. We expect that in the future, in addition to cervical cytology, more advanced techniques, including HPV genotyping, will be used to identify and triage those women most likely to harbor a clinically significant cervical lesion. Assays for HPV viral load and mRNA detection may be useful in both the triage of abnormal cervical cytology, and detecting persistent infection, which is associated with an increased risk for disease progression. We also reviewed new applications of technologies such as FISH and the detection of DNA methylation; although their clinical utility is still under investigation, they have the potential to provide valuable information in the identification of disease, risk of disease progression, and clinical management of patients. Although cervical cancer biomarkers will provide increasingly detailed and important information in countries that have organized screening programs, their utility will depend on the resolution of social and economic factors that have precluded the utilization of cervical cancer screening programs in developing nations. 

## References

[1] World Health Organization (2006). *Comprehensive Cervical Cancer Control: A Guide to Essential Practice*.

[2] (2006). Stat bite: worldwide cervical and uterine cancer incidence and mortality, 2002. *Journal of the National Cancer Institute*.

[3] Jemal A, Siegel R, Xu J, Ward E (2010). Cancer statistics, 2010. *CA Cancer Journal for Clinicians*.

[4] Gustafsson L, Pontén J, Zack M, Adami HO (1997). International incidence rates of invasive cervical cancer after introduction of cytological screening. *Cancer Causes and Control*.

[5] Schiffman M, Solomon D (2003). Findings to date from the ASCUS-LSIL Triage Study (ALTS). *Archives of Pathology and Laboratory Medicine*.

[6] Schiffman MH, Bauer HM, Hoover RN (1993). Epidemiologic evidence showing that human papillomavirus infection causes most cervical intraepithelial neoplasia. *Journal of the National Cancer Institute*.

[7] Walboomers JM, Jacobs MV, Manos MM (1999). Human papillomavirus is a necessary cause of invasive cervical cancer worldwide. *Journal of Pathology*.

[8] Wolf JK, Ramirez PT (2001). The molecular biology of cervical cancer. *Cancer Investigation*.

[9] Zur Hausen H (1999). Papillomaviruses in human cancers. *Proceedings of the Association of American Physicians*.

[10] Dunne EF, Unger ER, Sternberg M (2007). Prevalence of HPV infection among females in the United States. *Journal of the American Medical Association*.

[11] Schiffman M, Castle PE, Jeronimo J, Rodriguez AC, Wacholder S (2007). Human papillomavirus and cervical cancer. *Lancet*.

[12] Schiffman M (2007). Integration of human papillomavirus vaccination, cytology, and human papillomavirus testing. *Cancer*.

[13] Schlecht NF, Kulaga S, Robitaille J (2001). Persistent human papillomavirus infection as a predictor of cervical intraepithelial neoplasia. *Journal of the American Medical Association*.

[14] Stoler MH, Schiffman M (2001). Interobserver reproducibility of cervical cytologic and histologic interpretations: realistic estimates from the ASCUS-LSIL Triage Study. *Journal of the American Medical Association*.

[15] Doorbar J (2007). Papillomavirus life cycle organization and biomarker selection. *Disease Markers*.

[16] Stoler MH (2003). Human papillomavirus biology and cervical neoplasia: implications for diagnostic criteria and testing. *Archives of Pathology and Laboratory Medicine*.

[17] Dehn D, Torkko KC, Shroyer KR (2007). Human papillomavirus testing and molecular markers of cervical dysplasia and carcinoma. *Cancer*.

[18] Bulkmans NWJ, Rozendaal L, Snijders PJ (2004). POBASCAM, a population-based randomized controlled trial for implementation of high-risk HPV testing in cervical screening: design, methods and baseline data of 4402 women. *International Journal of Cancer*.

[19] Mayrand MH, Duarte-Franco E, Rodrigues I (2007). Human papillomavirus DNA versus Papanicolaou screening tests for cervical cancer. *New England Journal of Medicine*.

[20] Naucler P, Ryd W, Törnberg S (2007). Human papillomavirus and Papanicolaou tests to screen for cervical cancer. *New England Journal of Medicine*.

[21] Davies P, Kornegay J, Iftner T (2001). Current methods of testing for human papillomavirus. *Best Practice and Research: Clinical Obstetrics and Gynaecology*.

[22] Wright TC, Schiffman M (2003). Adding a test for human papillomavirus DNA to cervical-cancer screening. *New England Journal of Medicine*.

[23] Khan MJ, Castle PE, Lorincz AT (2005). The elevated 10-Year risk of cervical precancer and cancer in women with human papillomavirus (HPV) type 16 or 18 and the possible utility of type-specific HPV testing in clinical practice. *Journal of the National Cancer Institute*.

[24] Johnson LR, Starkey CR, Palmer J (2008). A comparison of two methods to determine the presence of high-risk HPV cervical infections. *American Journal of Clinical Pathology*.

[25] Einstein MH, Martens MG, Garcia FA (2010). Clinical validation of the Cervista HPV HR and 16/18 genotyping tests for use in women with ASC-US cytology. *Gynecologic Oncology*.

[26] Bartholomew DA, Luff RD, Quigley NB, Curtis M, Olson MC (2011). Analytical performance of Cervista^®^ HPV 16/18 genotyping test for cervical cytology samples. *Journal of Clinical Virology*.

[27] Peyton CL, Gravitt PE, Hunt WC (2001). Determinants of genital human papillomavirus detection in a US population. *Journal of Infectious Diseases*.

[28] Snijders PJ, van den Brule AJ, Meijer CJ (2003). The clinical relevance of human papillomavirus testing: relationship between analytical and clinical sensitivity. *Journal of Pathology*.

[29] Gravitt PE, Peyton C, Wheeler C, Apple R, Higuchi R, Shah KV (2003). Reproducibility of HPV 16 and HPV 18 viral load quantitation using TaqMan real-time PCR assays. *Journal of Virological Methods*.

[30] Carcopino X, Henry M, Benmoura D (2006). Determination of HPV type 16 and 18 viral load in cervical smears of women referred to colposcopy. *Journal of Medical Virology*.

[31] Hudson JB, Bedell MA, McCance DJ, Laimins LA (1990). Immortalization and altered differentiation of human keratinocytes in vitro by the E6 and E7 open reading frames of human papillomavirus type 18. *Journal of Virology*.

[32] Molden T, Kraus I, Karlsen F, Skomedal H, Hagmar B (2006). Human papillomavirus E6/E7 mRNA expression in women younger than 30 years of age. *Gynecologic Oncology*.

[33] Molden T, Nygård JF, Kraus I (2005). Predicting CIN2^+^ when detecting HPV mRNA and DNA by PreTect HPV-Proofer and consensus PCR: a 2-year follow-up of women with ASCUS or LSIL Pap smear. *International Journal of Cancer*.

[34] Birner P, Bachtiary B, Dreier B (2001). Signal-amplified colorimetric in situ hybridization for assessment of human papillomavirus infection in cervical lesions. *Modern Pathology*.

[35] Melsheimer P, Kaul S, Dobeck S, Bastert G (2003). Immunocytochemical detection of HPV high-risk type L1 capsid proteins in LSIL and HSIL as compared with detection of HPV L1 DNA. *Acta Cytologica*.

[36] Griesser H, Sander H, Hilfrich R, Moser B, Schenck U (2004). Correlation of immunochemical detection of HPV L1 capsid protein in pap smears with regression of high-risk HPV positive mild/moderate dysplasia. *Analytical and Quantitative Cytology and Histology*.

[37] Hilfrich R, Hariri J (2008). Prognostic relevance of human papillomavirus L1 capsid protein detection within mild and moderate dysplastic lesions of the cervix uteri in combination with p16 biomarker. *Analytical and Quantitative Cytology and Histology*.

[38] Galgano MT, Castle PE, Atkins KA, Brix WK, Nassau SR, Stoler MH (2010). Using Biomarkers as objective standards in the diagnosis of cervical biopsies. *American Journal of Surgical Pathology*.

[39] Zhang HS, Postigo AA, Dean DC (1999). Active transcriptional repression by the Rb-E2F complex mediates G1 arrest triggered by p16INK4a, TGF*β*, and contact inhibition. *Cell*.

[40] Keating JT, Cviko A, Riethdorf S (2001). Ki-67, cyclin E, and p16INK4 are complimentary surrogate biomarkers for human papilloma virus-related cervical neoplasia. *American Journal of Surgical Pathology*.

[41] Klaes R, Friedrich T, Spitkovsky D (2001). Overexpression of p16ink4a as a specific marker for dysplastic and neoplastic epithelial cells of the cervix uteri. *International Journal of Cancer*.

[42] Cuschieri K, Wentzensen N (2008). Human papillomavirus mRNA and p16 detection as biomarkers for the improved diagnosis of cervical neoplasia. *Cancer Epidemiology Biomarkers and Prevention*.

[43] Beauséjour CM, Krtolica A, Galimi F (2003). Reversal of human cellular senescence: roles of the p53 and p16 pathways. *EMBO Journal*.

[44] Tringler B, Gup CJ, Singh M (2004). Evaluation of p16INK4a and pRb expression in cervical squamous and glandular neoplasia. *Human Pathology*.

[45] Horn LC, Reichert A, Oster A (2008). Immunostaining for p16INK4a used as a conjunctive tool improves interobserver agreement of the histologic diagnosis of cervical intraepithelial neoplasia. *American Journal of Surgical Pathology*.

[46] Klaes R, Benner A, Friedrich T (2002). p16INK4a immunohistochemistry improves interobserver agreement in the diagnosis of cervical intraepithelial neoplasia. *American Journal of Surgical Pathology*.

[47] Zhang Q, Kuhn L, Denny LA, De Souza M, Taylor S, Wright TC (2007). Impact of utilizing p16INK4A immunohistochemistry on estimated performance of three cervical cancer screening tests. *International Journal of Cancer*.

[48] Bergeron C, Ordi J, Schmidt D, Trunk MJ, Keller T, Ridder R (2010). Conjunctive p16INK4a testing significantly increases accuracy in diagnosing high-grade cervical intraepithelial neoplasia. *American Journal of Clinical Pathology*.

[49] Murphy N, Ring M, Killalea AG (2003). p16INK4A as a marker for cervical dyskaryosis: CIN and cGIN in cervical biopsies and ThinPrep^*™*^ smears. *Journal of Clinical Pathology*.

[50] Samarawardana P, Dehn DL, Singh M (2010). p16^INK4a^ is superior to high-risk human papillomavirus testing in cervical cytology for the prediction of underlying high-grade dysplasia. *Cancer Cytopathology*.

[51] Denton KJ, Bergeron C, Klement P, Trunk MJ, Keller T, Ridder R (2010). The sensitivity and specificity of p16INK4a cytology vs HPV testing for detecting high-grade cervical disease in the triage of ASC-US and LSIL Pap cytology results. *American Journal of Clinical Pathology*.

[52] Kruse AJ, Baak JP, de Bruin PC (2001). Ki-67 immunoquantitation in cervical intraepithelial neoplasia (CIN): a sensitive marker for grading. *Journal of Pathology*.

[53] Kruse AJ, Baak JP, de Bruin PC, van de Goot FR, Kurten N (2001). Relationship between the presence of oncogenic HPV DNA assessed by polymerase chain reaction and Ki-67 immunoquantitative features in cervical intraepithelial neoplasia. *Journal of Pathology*.

[54] Singh M, Mockler D, Akalin A Dual localization of p16INK4a and Ki-67 detects high grade cervical intraepithelial neoplasia and cancer.

[55] Petry KU, Schmidt D, Scherbring S (2011). Triaging Pap cytology negative, HPV positive cervical cancer screening results with p16/Ki-67 Dual-stained cytology. *Gynecologic Oncology*.

[56] Schmidt D, Bergeron C, Denton KJ, Ridder R (2011). p16/ki-67 dual-stain cytology in the triage of ASCUS and LSIL papanicolaou cytology: results from the European equivocal or mildly abnormal Papanicolaou cytology study. *Cancer Cytopathology*.

[57] Liu H, Shi J, Wilkerson M (2007). Immunohistochemical detection of p16INK4a in liquid-based cytology specimens on cell block sections. *Cancer*.

[58] Eleutério J, Giraldo PC, Gonçalves AK (2007). Prognostic markers of high-grade squamous intraepithelial lesions: the role of p16INK4a and high-risk human papillomavirus. *Acta Obstetricia et Gynecologica Scandinavica*.

[59] Meyer JL, Hanlon DW, Andersen BT, Rasmussen OF, Bisgaard K (2007). Evaluation of p16INK4a expression in ThinPrep cervical specimens with the CINtec p16INK4a assay: correlation with biopsy follow-up results. *Cancer*.

[60] Wentzensen N, Bergeron C, Cas F, Vinokurova S, von Knebel Doeberitz M (2007). Triage of women with ASCUS and LSIL cytology: use of qualitative assessment of p16INK4a positive cells to identify patients with high-grade cervical intraepithelial neoplasia. *Cancer*.

[61] Bollmann R, Bollmann M, Henson DE, Bodo M (2001). DNA cytometry confirms the utility of the Bethesda System for the classification of Papanicolaou smears. *Cancer*.

[62] Bollmann R, Méhes G, Torka R, Speich N, Schmitt C, Bollmann M (2003). Human papillomavirus typing and DNA ploidy determination of squamous intraepithelial lesions in liquid-based cytologic samples. *Cancer*.

[63] Bollmann R, Méhes G, Speich N, Schmitt C, Bollmann M (2005). Aberrant, highly hyperdiploid cells in human papillomavirus-positive, abnormal cytologic samples are associated with progressive lesions of the uterine cervix. *Cancer*.

[64] Grote HJ, Nguyen HV, Leick AG, Böcking A (2004). Identification of progressive cervical epithelial cell abnormalities using DNA image cytometry. *Cancer*.

[65] Lorenzato M, Caudroy S, Nou JM (2008). Contribution of DNA ploidy image cytometry to the management of ASC cervical lesions. *Cancer*.

[66] Santin AD, Zhan F, Bignotti E (2005). Gene expression profiles of primary HPV16- and HPV18-infected early stage cervical cancers and normal cervical epithelium: identification of novel candidate molecular markers for cervical cancer diagnosis and therapy. *Virology*.

[67] Murphy N, Ring M, Heffron CC (2005). p16^INK4^A, CDC6, and MCM5: predictive biomarkers in cervical preinvasive neoplasia and cervical cancer. *Journal of Clinical Pathology*.

[68] Malinowski DP (2005). Molecular diagnostic assays for cervical neoplasia: emerging markers for the detection of high-grade cervical disease. *BioTechniques*.

[69] Siddiqui MT, Hornaman K, Cohen C, Nassar A (2008). ProEx C immunocytochemistry and high-risk human papillomavirus DNA testing in papanicolaou tests with atypical squamous cell (ASC-US) cytology: correlation study with histologic biopsy. *Archives of Pathology and Laboratory Medicine*.

[70] Shroyer KR, Homer P, Heinz D, Singh M (2006). Validation of a novel immunocytochemical assay for topoisomerase II-*α* and minichromosome maintenance protein 2 expression in cervical cytology. *Cancer*.

[71] Oberg TN, Kipp BR, Vrana JA (2010). Comparison of p16^INK4^a and ProEx^*™*^ C immunostaining on cervical ThinPrep^®^ cytology and biopsy specimens. *Diagnostic Cytopathology*.

[72] Conesa-Zamora P, Doménech-Peris A, Orantes-Casado FJ (2009). Effect of human papillomavirus on cell cycle-related proteins p16, Ki-67, cyclin D1, p53, and ProEx C in precursor lesions of cervical carcinoma: a tissue microarray study. *American Journal of Clinical Pathology*.

[73] Baylin SB, Ohm JE (2006). Epigenetic gene silencing in cancer—a mechanism for early oncogenic pathway addiction?. *Nature Reviews Cancer*.

[74] Belinsky SA, Nikula KJ, Palmisano WA (1998). Aberrant methylation of p16INK4a is an early event in lung cancer and a potential biomarker for early diagnosis. *Proceedings of the National Academy of Sciences of the United States of America*.

[75] Miranda E, Destro A, Malesci A (2006). Genetic and epigenetic changes in primary metastatic and nonmetastatic colorectal cancer. *British Journal of Cancer*.

[76] Schutte M, Hruban RH, Geradts J (1997). Abrogation of the Rb/p16 tumor-suppressive pathway in virtually all pancreatic carcinomas. *Cancer Research*.

[77] Yang HJ, Liu VW, Wang Y (2004). Detection of hypermethylated genes in tumor and plasma of cervical cancer patients. *Gynecologic Oncology*.

[78] Dong SM, Kim HS, Rha SH, Sidransky D (2001). Promoter hypermethylation of multiple genes in carcinoma of the uterine cervix. *Clinical Cancer Research*.

[79] Jeong DH, Youm MY, Kim YN (2006). Promoter methylation of p16, DAPK, CDH1, and TIMP-3 genes in cervical cancer: correlation with clinicopathologic characteristics. *International Journal of Gynecological Cancer*.

[80] Lin Z, Gao M, Zhang X (2005). The hypermethylation and protein expression of p16INK4A and DNA repair gene O6 -methylguanine-DNA methyltransferase in various uterine cervical lesions. *Journal of Cancer Research and Clinical Oncology*.

[81] Lea JS, Coleman R, Kurien A (2004). Aberrant p16 methylation is a biomarker for tobacco exposure in cervical squamous cell carcinogenesis. *American Journal of Obstetrics and Gynecology*.

[82] Kang S, Kim JW, Kang GH (2006). Comparison of DNA hypermethylation patterns in different types of uterine cancer: cervical squamous cell carcinoma, cervical adenocarcinoma and endometrial adenocarcinoma. *International Journal of Cancer*.

[83] Virmani AK, Muller C, Rathi A, Zoechbauer-Mueller S, Mathis M, Gazdar AF (2001). Aberrant methylation during cervical carcinogenesis. *Clinical Cancer Research*.

[84] Wong YF, Chung TK, Cheung TH (1999). Methylation of p16(INK4A) in primary gynecologic malignancy. *Cancer Letters*.

[85] Nehls K, Vinokurova S, Schmidt D (2008). p16 methylation does not affect protein expression in cervical carcinogenesis. *European Journal of Cancer*.

[86] Wentzensen N, Sherman ME, Schiffman M, Wang SS (2009). Utility of methylation markers in cervical cancer early detection: appraisal of the state-of-the-science. *Gynecologic Oncology*.

[87] Wang X, Zheng B, Li S (2009). Automated detection and analysis of fluorescent in situ hybridization spots depicted in digital microscopic images of Pap-smear specimens. *Journal of Biomedical Optics*.

[88] Heselmeyer-Haddad K, Sommerfeld K, White NM (2005). Genomic amplification of the human telomerase gene (TERC) in Pap smears predicts the development of cervical cancer. *American Journal of Pathology*.

[89] Heselmeyer K, Schröck E, du Manoir S (1996). Gain of chromosome 3q defines the transition from severe dysplasia to invasive carcinoma of the uterine cervix. *Proceedings of the National Academy of Sciences of the United States of America*.

[90] Allen DG, White DJ, Hutchins AM (2000). Progressive genetic aberrations detected by comparative genomic hybridization in squamous cell cervical cancer. *British Journal of Cancer*.

[91] Caraway NP, Khanna A, Dawlett M (2008). Gain of the 3q26 region in cervicovaginal liquid-based pap preparations is associated with squamous intraepithelial lesions and squamous cell carcinoma. *Gynecologic Oncology*.

[92] Seppo A, Jalali GR, Babkowski R (2009). Gain of 3q26: a genetic marker in low-grade squamous intraepithelial lesions (LSIL) of the uterine cervix. *Gynecologic Oncology*.

[93] Jarboe EA, Thompson LC, Heinz D, McGregor JA, Shroyer KR (2004). Telomerase and Human Papillomavirus as Diagnostic Adjuncts for Cervical Dysplasia and Carcinoma. *Human Pathology*.

